# Ampicillin and Ceftobiprole Combination for the Treatment of *Enterococcus faecalis* Invasive Infections: “The Times They Are A-Changin”

**DOI:** 10.3390/antibiotics12050879

**Published:** 2023-05-09

**Authors:** Simone Giuliano, Jacopo Angelini, Denise D’Elia, Monica Geminiani, Roberto Daniele Barison, Alessandro Giacinta, Assunta Sartor, Floriana Campanile, Francesco Curcio, Menino Osbert Cotta, Jason A. Roberts, Massimo Baraldo, Carlo Tascini

**Affiliations:** 1 Infectious Diseases Division, Department of Medicine, University of Udine and Azienda Sanitaria Universitaria Friuli Centrale, 33100 Udine, Italy; simone.giuliano@asufc.sanita.fvg.it (S.G.); carlo.tascini@asufc.sanita.fvg.it (C.T.); 2 Clinical Pharmacology and Toxicology Institute, University Hospital Friuli Centrale ASUFC, 33100 Udine, Italy; jacopo.angelini@asufc.sanita.fvg.it (J.A.);; 3 Department of Medicine, University of Udine (UNIUD), 33100 Udine, Italy; 4 Microbiology Unit, Udine University Hospital, 33100 Udine, Italy; 5 Department of Biomedical and Biotechnological Sciences, Section of Microbiology, University of Catania, 95123 Catania, Italy; 6 Institute of Clinical Pathology, Azienda Sanitaria Universitaria Friuli Centrale (ASUFC), 33100 Udine, Italy; 7 Faculty of Medicine, University of Queensland, Centre for Clinical Research (UQCCR), Brisbane, QLD 4029, Australia; 8 Herston Infectious Diseases Institute, Herston, QLD 4029, Australia; 9 Departments of Intensive Care Medicine and Pharmacy, Royal Brisbane and Women’s Hospital, Brisbane, QLD 4029, Australia; 10 Division of Anaesthesiology Critical Care Emergency and Pain Medicine, Nîmes University Hospital, University of Montpellier, 34095 Nîmes, France

**Keywords:** enterococcus infections, infective endocarditis, combination therapy, ceftobiprole, PBPs, therapeutic drug monitoring, ampicillin, TDM, enterococcus faecalis

## Abstract

Background: *Enterococcus faecalis* is responsible for a large variety of severe infections. This study is a case series reporting our experience in the treatment of *E. faecalis* invasive infections with ampicillin in combination with ceftobiprole (ABPR). Methods: We retrospectively analyzed all the medical records of patients admitted to the University Hospital of Udine from January to December 2020 with a diagnosis of infective endocarditis or primary or non-primary complicated or uncomplicated bacteremia caused by *E. faecalis*. Results: Twenty-one patients were included in the final analysis. The clinical success rate was very high, accounting for 81% of patients, and microbiological cure was obtained in 86% of patients. One relapse was recorded in one patient who did not adhere to the partial oral treatment prescribed. Therapeutic drug monitoring (TDM) was always performed for ampicillin and ceftobiprole, and serum concentrations of both drugs were compared to the MICs of the different enterococcal isolates. Conclusions: ABPR is a well-tolerated antimicrobial regimen with anti-*E. faecalis* activity. TDM can help clinicians optimize medical treatments to achieve the best possible efficacy with fewer side effects. ABPR might be a reasonable option for the treatment of severe invasive infections caused by *E. faecalis* due to the high level of enterococcal penicillin-binding protein (PBP) saturation.

## 1. Introduction

*Enterococcus faecalis* can cause a large variety of life-threatening invasive diseases, especially in the setting of heavy community-onset bacteremia arising in frail patients with a prosthetic heart valve, immunodepression, structural abnormalities of the urinary tract, urinary tract infections, and colorectal neoplasms [1,2,3,4]. Currently, the European Society of Cardiology (ESC) recommends ampicillin or amoxicillin in combination [5] with an aminoglycoside (AG) for the treatment of penicillin-susceptible, non-high-level aminoglycoside resistant (HLAR) *E. faecalis* infective endocarditis (IE), and a combination therapy of ampicillin or amoxicillin plus ceftriaxone (AC) for the treatment of penicillin-susceptible, non-HLAR and HLAR *E. faecalis* IE [6]. Because of literature reports of very high anti-enterococcal activity of ceftobiprole in vitro [7,8], and given the availability of therapeutic drug monitoring (TDM) for both ampicillin and ceftobiprole in our hospital, the choice of using ampicillin plus ceftobiprole combination (ABPR) [9] instead of AC for the treatment of IE and other invasive infections caused by *E. faecalis* has recently been increasing in our institution. Ampicillin is a well-known penicillin that covers a broad spectrum of infections, while ceftobiprole is a newer antibiotic that belongs to the fifth-generation class of cephalosporins, and it has a broad spectrum of antimicrobial activity against most Gram-positive and many Gram-negative bacteria. One of the most important features of ceftobiprole is represented by its activity against methicillin-resistant *Staphylococcus aureus* (MRSA) [10], which distinguishes this drug from the other cephalosporins and makes ceftobiprole a valid therapeutic option in some clinical conditions where older beta-lactams are not efficacious. Regarding dosing, ceftobiprole is approved for multiple administrations over an infusion period of two hours, every three hours, except in cases of mild and severe renal insufficiency, where a reduction in dosage is required. According to the latest ESC guidelines for the pharmacological treatment of *E. faecalis* IE, ampicillin should be prescribed at a total daily dose of 200 mg per kg by multiple intermittent intravenous administrations, with a time between each administration ranging between 4 and 6 h [6]. Since the TDM of both ampicillin and ceftobiprole is an in-house service, we routinely use it in our clinical practice for the management of the pharmacological antimicrobial therapy, and we can ultimately change the indicated dosage of the two beta-lactams promptly, thanks in part to reasonable turnaround times and the interpretation of the plasmatic concentrations of antibiotics by our clinical pharmacologists. Consequently, to maximize the time-dependent antimicrobial properties of ceftobiprole and ampicillin, we administer ceftobiprole by a prolonged infusion over three hours and ampicillin by continuous infusion, adjusting the dosage with respect to renal function. This therapeutic strategy aims to achieve the most effective pharmacodynamic and pharmacokinetic antimicrobial target represented by the highest percentage of plasmatic concentration of the antibiotic above the Minimum Inhibitory Concentration (MIC) (%T > MIC) during the interval between doses. At the same time, this method of drug delivery stabilizes the therapeutic antibiotic plasma level, preventing overexposure and the associated adverse events [11]. The present study is a case series of patients hospitalized because of invasive infections due to *E. faecalis* for whom an ABPR regimen was used. Our aim is to present the ABPR combination as a reasonable option for the treatment of *E. faecalis* invasive infections. So, we believe that the analysis performed in this paper might be of some interest to Infectious Disease and Internal Medicine specialists.

## 2. Results

Overall, 21 patients presented with an infection caused by *E. faecalis* that fulfilled the criteria of inclusion. Details about demographics, diagnosis of invasive enterococcal disease, duration of ABPR regimen, possible partial oral treatment, potential surgical intervention, and microbiological and clinical outcomes are provided in Table 1. A total of 13 out of 21 patients (61%) were diagnosed with IE. All cases of IE were left-sided. Among patients with IE, periannular extension and abscess were observed in three cases, all with prosthetic valve endocarditis. Furthermore, cardioembolic events were found in two patients (one with prosthetic aortic valve IE with embolization to spleen and kidney, the other one with native aortic valve IE with embolization to spleen only). Most patients (13/21, 61%) were admitted to the Internal Medicine ward; the remaining patients were hospitalized in a Cardiology/Cardiac Surgery ward. For 12 out of 21 patients (57%), a colonic lesion was identified through a colonoscopy as the portal of entry of enterococcal bacteremia. In five patients, a creatinine clearance ≥130 mL/min (i.e., augmented renal clearance, ARC) was demonstrated. For all *E. faecalis* isolates, ampicillin MIC was determined by broth microdilution using a Mueller–Hinton broth medium and a standard inoculum of 5 × 10^5^ colony-forming units (CFUs). MICs were read at the lowest concentration of the agent that completely inhibited visible growth, and susceptibility interpretations were established according to current European Committee on Antimicrobial Susceptibility Testing (EUCAST) breakpoints. Ampicillin MIC_50_ and MIC_90_ were 0.5 mg/L and 2 mg/L, respectively. Ceftobiprole MIC was determined using an MIC test strip (E-test). Ceftobiprole MIC_50_ and MIC_90_ were 0.5 mg/L and 1 mg/L, respectively.

Ceftobiprole and ampicillin were prescribed according to the Estimated Glomerular Filtration Rate (eGFR), so ceftobiprole was administered at the dose of 500 mg in eight-hourly intervals for fifteen patients; three patients received 500 mg of ceftobiprole in twelve-hourly intervals; the remaining three patients were administered 350 mg of ceftobiprole in eight-hourly intervals, 250 mg in twelve-hourly intervals, and 250 mg once daily, respectively. Ampicillin dose ranged between 4 g and 16 g daily by continuous infusion. Among patients with IE, the mean duration of the ABPR regimen was 27.8 ± 14.5 days. In patients with *E. faecalis* bacteremia, the mean duration of ABPR treatment was 20.4 ± 11.1 days. The TDM of ampicillin and ceftobiprole was always performed after the achievement of steady-state concentrations, and plasmatic concentrations of both of them were compared to the MICs of the different enterococcal isolates. Except for one patient for whom indefinite chronic suppressive therapy was administered, the mean duration of partial oral treatment was 19.0 ± 10.6 days. The average time to negative blood cultures was 7.8 days ± 4.2 days. No patient developed a breakthrough infection during the hospital stay. Two patients (2/21, 9%) experienced ABPR-related side effects such as seizure and a skin rash, respectively. For both of them, the ABPR was discontinued by the treating physician. A surgical procedure was performed on nine patients: eight of them (38%) underwent valve replacement, and one patient was managed with valve repair. The mean time to surgery was 18.4 ± 11.3 days. The mean length of hospital stay was 57.6 ± 44.3 days. Four patients (19%) were admitted to the Intensive Care Unit, and two of them with IE underwent orotracheal intubation. The three patients who died suffered from IE: one of them declined the surgical procedure, another one died before the surgical procedure, and the last one died immediately after cardiac surgery.

## 3. Discussion

Microorganisms that make up the genus *Enterococcus* are Gram-positive facultative anaerobic cocci that can exist as single cells, pairs (diplococci), short chains, and long chains and are difficult to differentiate morphologically from streptococci [12]. Enterococci are generally α-hemolytic or γ-hemolytic but may also appear to be β-hemolytic [12]. Most enterococci belong to Lancefield group D, with a minority of them belonging to Lancefield group Q; some of them are also motile [12]. Many enterococcal species are isolated in human infections, yet *E. faecalis* and *E. faecium* are able to cause the vast majority of clinical diseases [12]. Enterococci are the third most common cause of IE [13] and one of the leading causes of community-onset and nosocomial bloodstream infections (BSIs), accounting for 6% and 16% of cases, respectively [14]. IE caused by *E. faecium* is somehow rare [15], whereas the ratio of non-EI bacteremia cases to EI cases for *E. faecalis* is 1.2 to 1 [16]. This might be due to the lack of virulence factors such as hemolysin, gelatinase, and aggregation proteins in non-*E. faecalis* enterococci [17,18,19]. In addition, adhesin of collagen from enterococci (Ace), which is a microbial surface component recognizing adhesive matrix molecule that might play a pivotal role in heart valve binding, has been recognized in *E. faecalis* and has not been demonstrated in *E. faecium* [17,18,19,20].

Like in cases of IE caused by other bacterial and fungal [21] pathogens, IE due to *E. faecalis* is a biofilm-related disease. The *ebp* (encoding endocarditis and biofilm-associated pili) operon and *srtC* play a crucial role in biofilm formation by *E. faecalis*. It is possible that the assembly of pili ensues from cross-linking of precursor proteins such as EbpA, EbpB, and EpbC that are recognized by a specific sortase, SrtC; pili are involved in cell-surface interactions in the multi-stage process of biofilm building [22]. Risk factors for the acquisition of severe enterococcal infection include glucocorticoid treatment, immunosuppressive drugs, diabetes mellitus, chronic renal disease, HIV infection, solid organ and bone marrow transplantation, and malignancy [23]. Almost two-thirds of our patients had a gastrointestinal lesion identified at the endoscopic examination as the portal of entry of the enterococcal infection; these data are consistent with a retrospective analysis of Pericàs and co-workers reporting a strong relationship between *E. faecalis* IE and colorectal neoplasms [4]. Tolerance to penicillin that is clinically defined by a MIC/MBC (minimal bactericidal concentration, i.e., the minimal antibiotic concentration which causes 99.9% killing of the initial inoculum) ratio ≥32 [17] is a critical determinant of in vivo enterococcal reaction to beta-lactam therapy [24]. *E. faecalis* is generally four-fold more susceptible to aminopenicillins and ureidopenicillins than penicillin [12,25]. Moreover, it is characterized by a relative impermeability to aminoglycoside antibiotics; hence, huge and potentially toxic serum concentrations of aminoglycosides are required to achieve adequate intracellular concentration for binding to the ribosomal lethal target [26]. Nonetheless, increased permeability resulting from bacterial cell exposure to an agent with activity against the cell wall, such as penicillin, ampicillin, or a glycopeptide, allows for higher intracellular aminoglycoside concentration to be reached, thus triggering a bactericidal effect [26]. Traditionally, therapeutic regimens for the treatment of severe *E. faecalis* infections have been based on the combination of penicillin or ampicillin plus an aminoglycoside in order to achieve bactericidal activity. Moreover, encouraging data on the efficacy of vancomycin against vancomycin susceptible-*E. faecalis* have also been described in the real world [27]. Monotherapy is generally non-bactericidal; hence, life-threatening infections such as endocarditis and bacteremia might require combination therapy [28]. In the setting of infective endocarditis (IE) caused by ampicillin-and gentamicin-susceptible strains, a treatment based on AG was previously suggested [29]. In 1995, Mainardi and co-workers demonstrated synergism between amoxicillin and cefotaxime through partial saturation of low-molecular-weight penicillin-binding proteins (PBPs) 4 and 5 by amoxicillin and the total saturation of high-molecular-weight PBPs 2 and 3 by cefotaxime [30]. This study inferred that in clinical strains of *E. faecalis*, low-molecular-weight PBP4 and PBP5 play an essential role in cell growth, and the non-essential PBP2 and PBP3 might be engaged in cell wall assembly when the low-molecular-weight PBPs are out of order because of beta-lactam inhibition [31]. Based on these findings, Galvaldà [32] et al. in 2007 and Fernandez-Hidalgo [33] et al. in 2013 observed that the combination of AC was as effective as AG for the treatment of non-HLAR and HLAR *E. faecalis* IE [34]. AC showed similar effectiveness compared to AG with lower rates of side effects such as ototoxicity and nephrotoxicity [32,33,34]. In the last three years, a growing number of patients hospitalized in our Institution with severe *E. faecalis* infections have been treated with ABPR. The rationale behind this choice has been based on the following reasons:

1. Ceftobiprole medocaril is a new cephalosporin approved for community-acquired pneumonia and nosocomial pneumonia (including ventilated hospital-acquired pneumonia but not ventilator-acquired pneumonia) with very low cumulative *E. faecalis* MIC percent distribution (MIC_90_ 4 mg/L) [7]. Studies of clinical pharmacology demonstrated a 99% probability of PK-PD target attainment up to a MIC of 4 mg/L for Gram-positive cocci for the 500 mg 8-hourly dosing regimen approved for the aforementioned indications [35].
2. Similarly to ceftriaxone [36], ceftobiprole seems to penetrate adequately into heart valve tissues, as demonstrated by Boni and co-workers, who found ceftobiprole valve concentrations of 2.26 (IQR 2.14–2.69) µg/g in a patient with mitral native valve IE who underwent surgery [37].
3. An epidemiologic analysis of the populations of *E. faecalis* isolated in our Institution showed a ceftobiprole MIC distribution constantly ≤2 mg/L.
4. As a member of the pyrrolidinone-3-ylidene methyl cephems, ceftobiprole binds with high affinity to the *E. faecalis* PBPs [8]. Unlike ceftriaxone, ceftobiprole exhibits the ability to inhibit non-essential high-molecular-weight enterococcal PBPs [38,39] and maintains a higher affinity for the low-molecular-weight essential PBP4 [40]. PBP4 is a very critical lethal target and is the main determinant of beta-lactam sensitivity in *E. faecalis*; conformational alteration of this enzyme may be responsible for reduced beta-lactam susceptibility due to alteration of its catalytic motif [40,41,42,43], although hyperexpression of *pbp4* gene and mutations in PBP4 amino acid sequence may be responsible for reduced beta-lactam *E. faecalis* susceptibility, this seems not to be the case for ceftobiprole bactericidal activity that remains unaffected [40].
5. An epidemiologic analysis of the populations of *E. faecalis* isolated in our Institution showed an ampicillin MIC distribution constantly ≤2 mg/L.
6. A pharmacologic study by Arensdorff and colleagues showed the achievement of a mean plasma amoxicillin concentration of 18.5 mg/L after administration of the drug by continuous intravenous infusion [43].
7. Ceftobiprole, in combination with amoxicillin, showed a synergistic and bactericidal effect against *E.faecalis* [44,45].
8. In our hospital, ceftobiprole and ampicillin TDM is available for proper drug dosing.

In agreement with these assumptions, we have been increasingly using the ABPR combination, and we have been monitoring the plasmatic concentrations of ampicillin and ceftobiprole to exploit their pharmacological properties and to achieve the pharmacokinetic and pharmacodynamic targets. The TDM that we performed in the patients of this case series allowed us to measure both ampicillin and ceftobiprole plasmatic concentrations. Taking into consideration the measured antibiotic concentrations, we adjusted the dosing regimen of the two beta-lactams according to their PK and PD properties in relation to the corresponding measured MICs and to the concomitant pathophysiological variations, paying special attention to the estimated renal clearance of both ampicillin and ceftobiprole. To our knowledge, this approach is poorly described in the literature, and these data might provide further evidence supporting this pharmacological approach. Indeed, beta-lactam antibiotics such as ampicillin and ceftobiprole are time-dependent drugs; hence, their therapeutic efficacy in terms of maximum bacterial killing is linked to the time the free drug concentration stands above the MIC [46]. For penicillins and cephalosporins, a time above the MIC ≥ 40–50% and 60–70% of the dosing interval, respectively, is associated with bacteriologic cure [46,47,48]. TDM is even more essential in the setting of IE. Antibiotics penetrate poorly into the core of the vegetation in which drug concentration is supposed to be stable and in balance with the plasmatic concentration; thus, optimizing dose by means of TDM and, therefore, adjusting plasmatic and core intra-vegetation concentrations helps achieve microbiological and clinical cure [49,50]. Consistently, the clinical success rate was very high, accounting for 81% of patients, and microbiological cure was obtained in 86% of patients. The time to bacteremia clearance was 7.8 days ± 4.2 days, but this information is not relevant because FUBCs were not obtained on scheduled time. Favorable microbiological outcomes were observed even in five patients with ARC, a potential source of drug underexposure in critically ill patients, such as those with burns or sepsis [46]. This result shows that appropriate ampicillin and ceftobiprole dosing regimens were administered to our patients with severe enterococcal infections and ARC. Another possible speculative explanation for the excellent clinical and microbiological outcomes that we observed is the presumable activity of ABPR on mature biofilm. IE caused by *E. faecalis* is a biofilm-associated disease [51]. A biofilm is a community of bacteria that adhere to inert, dead, or living surfaces and are embedded in a hydrated polymeric matrix of their own synthesis [52]. Biofilm-associated infections are difficult to treat because of the slow diffusion of antibiotics in the core of the matrix [53] and the poor penetration of antibodies or innate immunity-related solutes [52]. Biofilms act as “niduses” of active infection because of the programmed disengagement of planktonic cells [54]; in addition, the reduced vulnerability to antibiotics of cells in biofilms might be related to the fact that some bacteria within the biofilm are malnourished, and as a result, they exist in a state of slow growth or starvation [55]. We could assume that another possible effect of slow bacterial growth inside biofilm is the reduced expression of PBPs, which are involved in cell wall building [56]. In planktonic and biofilm cultures of *E. faecalis*, Thieme and colleagues have found that while combining cephalosporins or gentamicin with ampicillin might be advantageous in treating persistent bacteremia caused by planktonic cells, it does not appear to be superior to monotherapy against mature biofilms [55]. Additionally, higher doses of ceftriaxone, such as those used in combination therapy with ampicillin, may even be harmful, as high cephalosporin concentrations seem to favor the selection of enterococcal small colony variants (i.e., antibiotic-resistant morphologic variants that grow into tiny colonies [57] and are the results of the intrinsic bacterial ability to survive anti-infective drugs) [58]. This could not be the case for ampicillin combinations with ceftobiprole because of the wider spectrum of PBPs bound with greater affinity by the latter compared to ceftriaxone. Indeed, ceftobiprole exhibited promising efficacy against biofilms formed by both methicillin-susceptible and -resistant staphylococci, either alone or in combination with other agents [59] and, perhaps, a similar effect could also be postulated for *E. faecalis* infections. This concept might be of significant importance if we take into consideration that the vast majority of *E. faecalis* IE that we observed in a twelve-month period in our hospital were prosthetic valve IE. Indeed, the process of biofilm formation is more important for prosthetic valve IE than for native valve IE [21]. One relapse was recorded in one patient who did not adhere to the partial oral treatment prescribed. Concerning partial oral treatment, the most commonly administered regimen was a combination of amoxicillin-clavulanic acid and cefditoren pivoxil. This choice was based on the fact that cefditoren pivoxil has a high affinity for *Streptococcus pneumoniae* PBP2X, which displays some similarities to *E. faecalis* PBP4; this might justify a certain putative activity of cefditoren pivoxil against *E. faecalis* [60,61]. This study is a case series and, therefore, presents several limitations. First, the lack of a control group means that it is not possible to demonstrate that outcomes depended on ABPR and were not influenced by other factors, such as patient features. Secondly, this case series, as such, is conditioned by selection bias and measurement bias, and patients included in the analysis might not reflect the characteristics of the general population. Moreover, it is a single-center study. Finally, the retrospective design of this study and the limited number of cases we described gives this study a lower level of evidence.

## 4. Materials and Methods

### 4.1. Study Design, Patients, Definitions

Medical data of all patients >18 years of age admitted to the University Hospital of Udine because of an invasive infection due to *E. faecalis* during the period from January to December 2020 were retrospectively evaluated. The need for written informed consent was waived due to the retrospective design. Criteria of inclusion were as follows: (1) diagnosis of primary or non-primary uncomplicated or complicated bacteremia; (2) diagnosis of infective endocarditis (IE); (3) administration of combination antimicrobial chemotherapy with ampicillin and ceftobiprole (ABPR) for at least half of the total intravenous treatment period; and (4) therapeutic drug monitoring (TDM) data were available for both ampicillin and ceftobiprole. The plasmatic concentration of ampicillin was measured by means of high-performance liquid chromatography coupled with ultraviolet (HPLC-UV), whereas the plasmatic concentration of ceftobiprole was measured by ultra-high performance liquid chromatography (UHPLC).

Information extracted from medical charts included demographics, predisposing conditions, biochemical and microbiological data, inpatient and outpatient antibiotic regimens, breakthrough infections, length of hospital stay, and outcome. Primary bacteremia was defined as a bloodstream infection without evidence of an apparent portal of entry [62]. Uncomplicated bacteremia was defined as (i) negative results of follow-up blood culture at 48 h to 96 h days after index bacteremia, (ii) defervescence within 72 h of treatment, and (iii) no demonstration of metastatic infection or IE 12. The diagnosis of IE was made according to modified Duke criteria [63]. Based on the presence or absence of foreign intracardiac material, IE was defined as prosthetic valve EI and native valve EI, respectively [63]. A periannular abscess was defined as a region of necrosis containing purulent material with no extension to the cardiovascular lumen, as observed at direct inspection during the surgical procedure [64]. A breakthrough infection was defined as evidence of enterococcal infection during the administration of an antimicrobial regimen which was considered likely active against E. faecalis. Partial oral treatment [65] was defined as outpatient oral therapy with likely anti-enterococcal activity, which was administered for half or less than half the total antimicrobial treatment after ABPR. Microbiological eradication was defined as negative follow-up blood cultures (FUBCs) after the index positive blood culture at some point during ABPR treatment and negative valve culture in patients who underwent surgery. Time to negative blood cultures was defined as the time of bacteremia clearance as documented by FUBCs. Time to surgery was defined as the time interval between the first positive blood culture and the surgical procedure when performed. Outcome was defined in terms of mortality at 28 days after the diagnosis of the infection and relapse (IE caused by the same microorganism [66]).

### 4.2. Statistical Analysis and Measures

Because of the nature of this study and the lack of comparison groups, we performed only a descriptive analysis in which we summarized the main characteristics of the patients included in the case series to simplify the data on the clinically relevant outcomes of interest from our sample. Consequently, no inferential statistics were performed. Demographic and clinical variables were described as mean ± standard deviation.

## 5. Conclusions

ABPR is a well-tolerated antimicrobial regimen with anti-*E. faecalis* activity. Moreover, when available, TDM can help clinicians optimize medical treatments to achieve the best possible efficacy with fewer side effects. Data on ceftobiprole use in this type of infection are lacking, but in our opinion, ABPR might be a reasonable option for the treatment of severe invasive infections caused by *E. faecalis*; the achievement of high levels of PBPs saturation might establish a bactericidal effect against the pathogen. Importantly, ceftobiprole might have enhanced activity against biofilms, and this could be of great significance for the treatment of biofilm-associated diseases such as IE or other intravascular infections. This new therapeutic approach could be useful, especially considering the increasing problem of antimicrobial resistance. Combination therapy of ampicillin and ceftobiprole could provide an additional pharmacologic option to augment the limited anti-infective treatments available in this clinical setting with a favorable balance between efficacy and safety. Further prospective and appropriately designed studies are needed in order to better characterize this antibiotic combination, not only in terms of its pharmacodynamic and pharmacokinetic properties, but also in terms of its effect on relevant clinical outcomes and its potential ecological impact.

## Figures and Tables

**Table 1:** Clinical features and outcomes of 21 patients with *E. faecalis* infection treated with the combination of ampicillin plus ceftobiprole.

Patient Number; Sex; Age	Diagnosis	Duration ABPR (Days)	Partial Oral Treatment Duration after ABPR (Days)	Surgery	Microbiological Eradication	Clinical Outcome
1; male; 41	IE (prosthetic aortic valve endocarditis)	8	linezolid; 20	Valve replacement with bioprosthesis	yes	cure
2; male; 80	IE (prosthetic aortic valve endocarditis)	30	amoxicillin/ clavulanate cefditoren; 37	Valve replacement with bioprosthesis	no	cure
3; female; 79	IE (prosthetic aortic valve endocarditis)	22	/	Valve replacement with bioprosthesis	no	cure
4; male; 83	IE (prosthetic aortic valve endocarditis)	5	/	/	yes	death
5; male; 52	IE (native aortic and mitral valve endocarditis)	19	amoxicillin /clavulanate cefditoren; 20	Valve replacement with bioprosthesis	yes	relapse
6; male; 62	IE (native mitral valve endocarditis)	33	amoxicillin /clavulanate cefditoren; 13	Mitral valvuloplasty	yes	cure
7; male; 64	IE (native aortic valve endocarditis)	Not known	/	Valve replacement with bioprosthesis	yes	cure
8; female; 81	IE (prosthetic aortic valve endocarditis and spondylodiscitis)	40	/	/	yes	cure
9; male; 75	Primary, complicated bacteremia (aortic vascular graft infection)	23	/	/	yes	cure
10; male; 82	IE (prosthetic aortic valve endocarditis)	43	amoxicillin/ clavulanate cefditoren; 5	Valve replacement with bioprosthesis	yes	cure
11; male; 80	IE (native mitral valve endocarditis)	21	/	/	yes	death
12; male; 83	IE (prosthetic aortic valve endocarditis)	25	/	/	yes	cure
13; female; 61	IE (native aortic valve endocarditis)	27	/	Valve replacement with bioprosthesis	yes	cure
14; female; 85	IE (prosthetic aortic and native mitral valve endocarditis)	60	/	Valve replacement with bioprosthesis	no	death
15; male; 76	Primary, uncomplicated bacteremia	14	/	/	yes	cure
16; female; 68	Primary, complicated bacteremia (septic arthritis)	46	/	/	yes	cure
17; female; 84	Primary, uncomplicated bacteremia	14	/	/	yes	cure
18; male; 60	Primary, uncomplicated bacteremia	27	/	/	yes	cure
19; male; 27	Primary, uncomplicated bacteremia	12	/	/	yes	cure
20; female; 68	Primary, complicated bacteremia (vertebral osteomyelitis)	17	amoxicillin/ clavulanate cefditoren; indefinite	/	yes	cure
21; male; 74	Primary, complicated bacteremia (vertebral osteomyelitis)	10	/	/	yes	cure

Footnotes: IE, infective endocarditis; ABPR, ampicillin plus ceftobiprole combination therapy.

## Data Availability

The data presented in this study are available on request from the corresponding author. The data are not publicly available due to restrictions e.g., privacy or ethical.
